# Intrapulmonary myelolipoma and its computed tomography features: A case report and literature review

**DOI:** 10.3892/ol.2015.2913

**Published:** 2015-01-28

**Authors:** QINGQING XU, XINDAO YIN, WENBIN HUANG, JUN SUN, XINYING WU, LINGQUAN LU

**Affiliations:** 1 Department of Radiology, Huai’an First People’s Hospital, Nanjing Medical University, Huai’an, Jiangsu 223300, P.R. China; 2 Department of Radiology, Nanjing Medical University Affiliated Nanjing Hospital, Nanjing, Jiangsu 210006, P.R. China; 3 Department of Pathology, Nanjing Medical University Affiliated Nanjing Hospital, Nanjing, Jiangsu 210006, P.R. China

**Keywords:** myelolipoma, lung, computed tomography, pathology

## Abstract

Intrapulmonary myelolipoma is a rare, benign tumor composed of mature adipose tissue and normal hematopoietic cells. To the best of our knowledge, 10 cases of intrapulmonary myelolipoma, including the present case, have been reported to date, and the majority have focused on the pathological diagnosis of the disease. The radiological features of intrapulmonary myelolipoma have not been studied. Therefore, the present study reports a case of primary myelolipoma in the lung, and examines its computed tomography features and pathology. Furthermore, other potential diagnoses are discussed in the context of the relevant literature. The present report describes the case of a 57-year-old female who experienced chills, but no coughing or expectoration, with an intermittent fever of 38.6°C that had been apparent for 13 days. Chest CT scan revealed a benign nodule and bronchiectasis in the lower lobe of the right lung. The patient then underwent a lobectomy of the lower right lung by thoracoscopy. The histological analysis of the excised specimen identifid a myelolipoma consisting of mature adipose tissue and hematopoietic cells. There was no recurrence after 513 days of follow-up, as shown by CT.

## Introduction

Myelolipoma is a rare and benign tumor that is typically asymptomatic. The tumor most frequently develops in the adrenal gland, and consists of mature adipose tissue and normal hematopoietic cells (1). Extra-adrenal myelolipomas are extremely rare, particularly in the presacral or perineal space (2). Intrapulmonary myelolipoma is relatively rare; up to now, only 10 cases of intrapulmonary lesions have been reported worldwide (3–11). Conservative resection strategies may be appropriate, as recurrence and malignancy have not been reported. In the present study, a case of intrapulmonary myelolipoma is described, and the computed tomography (CT) findings and potential confounding characteristics that may lead to incorrect diagnoses are reviewed. Informed consent was obtained from the patient.

## Case report

### Patient presentation

A 57-year-old female was admitted to the Nanjing Medical University Affiliated Nanjing Hospital (Nanjing, Jiangsu, China), with an intermittent fever of 38.6°C that had been apparent for 13 days. The patient experienced chills, but no coughing or expectoration. A chest CT scan performed at a local hospital revealed the presence of a nodule, bronchiectasis and an infection in the lower lobe of the right lung. Subsequent to treatment with 3,200,000 IU of patulin and antibiotics every 8 h for 5 days (orally), the patient underwent a follow-up chest CT scan at the Nanjing Medical University Affiliated Nanjing Hospital, which revealed a benign nodule and bronchiectasis in the lower lobe of the right lung. The tumor was observed in the tributary of the lower right pulmonary vein, but had not altered in size during the time between the two CT scans. The results of routine blood work appeared within normal limits. The patient then underwent a lobectomy of the lower right lung by thoracoscopy. The histological analysis of the excised specimen identified a myelolipoma consisting of mature adipose tissue and hematopoietic cells.

### CT procedures

A single-spiral CT scanner (Asteion; Toshiba Medical Systems, Tokyo, Japan) was used to perform the chest scan, using the following parameters: Tube voltage, 120 kV; tube current, 140 MA; window width, 1,700 Hounsfield units (HU); window level, -550 HU; and matrix size, 480×480 pixels. The CT slice thickness and interval were each 10 mm. The CT image was obtained from the apex to the base of the lung, with the patient at full end-inspiration and in the supine position.

### CT findings

**Figure 1:**
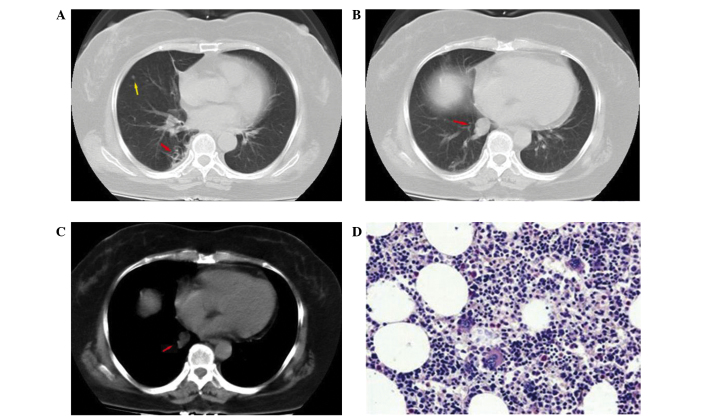
(A) Chest computed tomography (CT) (lung window). CT revealing bronchiectasis (red arrow) and small nodules (yellow arrow), with infection in the lower lobe of the right lung. (B) Chest CT (lung window). CT revealing a small nodule (red arrow) in the lower right lung. (C) Chest CT (mediastinal window). CT revealing a small nodule (red arrow) in the right lower lung, with the density of fat. (D) Hematoxylin and eosin staining revealing that the tumor was composed of mature adipose tissue and hematopoietic cells.

The axial CT images of the chest revealed a smooth and well-defined nodule of heterogeneous composition in the medial segment of the lower lobe of the right lung. The largest area of the nodule in the axial plane was ~14×15 mm. Negative density values were observed in the mass, with an average density of -46 HU (range, −30 to −68 HU). The mass was adjacent to the tributary of the lower right pulmonary vein. No density shadow, indicating calcification, was detected in the nodule by CT (Fig. 1A and B), and cystic bronchiectasis was present within the adjacent lung (Fig. 1C).

### Pathological findings

Grossly, the nodule was grayish-red and measured 16 mm in diameter at its largest point. Light microscopy revealed that the tumor was composed of mature adipose tissue and normal mature hematopoietic cells, including myeloid, erythroid and megakaryocytic cells, in normal proportions. No trabecular bone, calcification or ossification was observed in the tumor (Fig. 1D).

## Discussion

Myelolipoma was first described in the early 20th century, and is usually observed in the adrenal gland (1). The occurrence of extra-adrenal myelolipomas is less common, however, they have been observed in the presacral soft tissue, retroperitoneum, spleen, liver, stomach, mediastinum and nasal cavity (2,12–15). Intrapulmonary myelolipoma is rare, and to the best of our knowledge, only 10 cases (including three cases reported in the Chinese literature and the present case) have been documented (3–11). The patients in these cases ranged in age between 45 and 81 years, and were predominately male. Table 1 summarizes the patient and tumor characteristics of the reported intrapulmonary myelolipomas, including the data from the present study. A diagnosis of intrapulmonary myelolipoma is usually made when abnormal ratios of mature adipose tissue and hematopoietic cells, including myeloid, erythroid and megakaryocytic elements, and occasionally lymphocytes, are observed histologically (2,4,10,11,15).

In total, eight of the 10 previously reported cases of intrapulmonary myelolipoma were solitary, and two were multifocal. Intrapulmonary myelolipomas are usually small nodules measuring <30 mm at their largest dimension, however, one case revealed a nodule of 7 cm in length (15). Patients with intrapulmonary myelolipoma usually present with a history of pneumonia, however, one literature case presented with a bronchial carcinoid tumor (10), and the patient in the present study presented with a history of pneumonia and bronchiectasis. The CT features observed in the present case study were as expected, with the lesions typically possessing negative HU values from macroscopic fat. Due to the intermixed hematopoietic tissue, the attenuation of macroscopic fat is usually heterogeneous and higher than that of retroperitoneal fat, as was demonstrated in this patient. Regions of higher density, due to hemorrhage and calcification, would also be observed. The tumors may also be mildly or moderately enhanced following administration of intravenous contrast agents. Upon magnetic resonance imaging, lesions demonstrate high signal intensity from mature adipose on T1- and T2-weighted imaging. Fat suppression imaging also reduces signal intensity in the fatty components of lesions (16). In addition, myeloid elements demonstrate low signal intensity upon T1-weighted imaging, and intermediate intensity upon T2-weighted imaging.

CT reveals certain intrapulmonary myelolipomas to be centrally located masses, leading to obstructive pneumonia and atelectasis in the lobe or lung segment, and a frequent misdiagnosis of lung cancer. In these cases, accurate diagnoses are difficult, even following histological examination of the biopsy specimens obtained by bronchoscopy (3,6). Upon CT, negative HU values of adipose components should distinguish myelolipomas from lung cancer. Intrapulmonary myelolipomas located in the periphery of the lung, which demonstrate adipose densities upon CT, are usually small masses with smooth, heterogeneous and well-defined borders (6). This was the clinical presentation of the patient in the present study.

Intrapulmonary myelolipomas must be differentiated from phlebangiomas, hamartomas, lipomas and teratomas. Intrapulmonary phlebangiomas are rare tumors, which are often enhanced significantly following administration of intravenous contrast agents (17). In the present case study, without CT to identify the lipid component of the lesion, the nodule in the tributary of the lower-right pulmonary vein could potentially have been be misdiagnosed as a phlebangioma. Intrapulmonary hamartomas are common, benign masses in the pulmonary parenchyma. The histological makeup of these tumors is characterized by a fibromyxoid stroma, cartilage, bronchial cells, adipose tissue and bone. The characteristic ‘popcorn-like’ calcification that occurs with these masses is frequently observed by CT (18). Intrapulmonary lipomas are exceptionally rare. Only 10 cases have been reported to date in Medline, and no marked gender differences are evident with respect to these tumors (19,20). Pathologically, intrapulmonary lipomas are grossly observed as being well-defined, thinly-encapsulated and rounded, pale-yellow masses, which are composed of mature adipose tissue upon microscopic analysis (19). A homogeneous and defined lesion that contains a high density of lipid can be revealed by CT imaging. No contrast enhancement is observed with these masses, as they lack soft tissues (19,20). Myelolipomas that are predominantly composed of fatty tissue are difficult to distinguish from lipomas. Intrapulmonary teratomas may be malignant and upon histopathology, contain tissue that originates from any of the three germinal layers. Calcification is a typical radiological finding and is extremely valuable for clinical diagnoses when CT reveals discrete areas of soft tissue, fat, fluid or punctate calcification (20). In the case that peripheral myelolipomas in the lung are mainly composed of hematopoietic cells, it may be necessary to perform a percutaneous needle biopsy for a definite diagnosis.

In general, accurate identification of intrapulmonary myelolipoma depends on histopathology. However, it is important to understand the radiological characteristics, which can provide valuable diagnostic clues in clinical practice. Although it is difficult to achieve an accurate diagnosis, a chest CT scan demonstrating a well circumscribed nodule with low attenuation of adipose tissue is helpful in the diagnosis of intrapulmonary myelolipomas. Conservative resection strategies may be appropriate, as recurrence and malignancy have not been reported. However, given the likelihood of subsequent hemorrhage or compression of adjacent bronchi by myelolipomas, surgical removal is the ideal choice.

## Figures and Tables

**Table I:** Patient and tumor characteristics of reported intrapulmonary myelolipomas.

Case	First author/s (ref.)	Age, years/gender	Patient history	Tumor location	Number of sites	Size, cm	Diagnosis method
1	Saleeby (11)	81/F	Pneumonia	Peripherally	Single	ϕ2.5	Autopsy
2	Hunter *et al* (4)	70/F	RA, steroid	Peripherally	Multiple	ND	Biopsy
3	Ziolkowski *et al* (9)	49/M, 59/M	Pneumonia	LLL, RLL	Multiple	7×5×5, ϕ2	Autopsy, resection
4	Zunarelli *et al* (10)	52/M	MGUS, BC	RLL	Single	ND	Lobectomy
5	Sabate and Shahian (7)	54/M	HC	LUL	Single	ϕ2.5	Enucleation
6	Sato *et al* (8)	71/M	Lung cancer	LLL	Single	ϕ2.0	Autopsy
7	Lu and Xiao (6)	45/M	Pneumonia	LUL	Single	ϕ1.5	Lobectomy
8	Lin *et al* (5)	45/M	ND	LUL	Single	4.5×3.5×2.3	ND
9	Huang *et al* (3)	53/M	Pneumonia, atelectasis	LL	Single	2.3×1.2×1.0	Biopsy
10	Present case	57/F	Bronchiectasis	RLL	Single	ϕ1.6	Lobectomy

F, female; M, male; P, peripherally; RA, rheumatoid arthritis; MGUS, monoclonal gammopathy of undetermined significance; BC, bronchial carcinoid tumor; HC, hypercholesterolemia; LLL, left lower lobe; LUL, left upper lobe; ND, not determined; RLL, right lower lobe; RUL, right upper lobe; LL, left lung.
